# Association between stress hyperglycemia ratio and delirium in older hospitalized patients: a cohort study

**DOI:** 10.1186/s12877-022-02935-6

**Published:** 2022-04-04

**Authors:** Quhong Song, Miao Dai, Yanli Zhao, Taiping Lin, Li Huang, Jirong Yue

**Affiliations:** 1 Department of Geriatrics and National Clinical Research Center for Geriatrics, West China Hospital, Sichuan University, No. 37, Guo Xue Xiang, Chengdu, 610041 Sichuan China; 2 Jiujiang First People’s Hospital, Jiujiang, China

**Keywords:** Delirium, Stress hyperglycemia ratio, Hyperglycemia, Glycosylated hemoglobin, Older people

## Abstract

**Background:**

It remains unclear whether stress hyperglycemia is associated with delirium. We performed this cohort study to determine the association between stress hyperglycemia and delirium.

**Methods:**

We consecutively enrolled patients aged ≥70 years who were admitted to the Geriatric Department of West China Hospital between March 2016 and July 2017. Stress hyperglycemia ratio (SHR) was calculated as fasting blood glucose divided by estimated average glucose derived from glycosylated hemoglobin (HbA1c) and was classified into three tertiles. Delirium was screened within 24 h of admission and three times daily thereafter, using the confusion assessment method. The Cox proportional hazards models were used to assess the association of SHR with delirium.

**Results:**

Among 487 included patients (mean age 83.0 years, 72.0% male), 50 (10.3%) patients experienced delirium during hospitalization. Compared to the second tertile, both the lowest and the highest SHR tertiles were independently associated with delirium (hazard ratio [HR] 3.71, 95% confidence interval [CI] 1.45-9.51; and HR 2.97, 95% CI 1.29-6.81, respectively). Similar results were found after further adjusting for statin comedication. Multiple-adjusted restricted cubic splines revealed a nonlinear relationship between SHR and delirium (*P*_nonlinearity_=0.04). Adding SHR to conventional risk factors improved the risk prediction of delirium (net reclassification index 0.39, *P*=0.01; integrated discrimination improvement 0.07, *P*=0.03). Subgroup analyses indicated that the relationship between SHR and delirium was more apparent in patients with HbA1c <6.5%, with significantly higher HR in the first (3.65, 95% CI 1.11-11.97) and third (3.13, 95% CI 1.13-8.72) SHR tertiles compared to the second tertile, while there was no significant association between SHR and delirium in those with HbA1c ≥6.5%.

**Conclusions:**

Both lower and higher SHR were associated with increased risk of delirium but only in patients with HbA1c <6.5%. Admission SHR may serve as a promising predictor of delirium, and incorporating this biomarker into prediction algorithms might have potential clinical utility in aiding delirium risk stratification, especially in those with HbA1c <6.5%.

**Supplementary Information:**

The online version contains supplementary material available at 10.1186/s12877-022-02935-6.

## Background

Delirium, an acute neuropsychiatric syndrome characterized by fluctuating disturbance in attention and cognition, is a common complication in elderly hospitalized patients [1, 2]. It affects up to 50% of those older than 65 years [2] and is associated with multiple adverse outcomes, including prolonged hospitalization, functional disability, long-term cognitive impairment, and increased mortality [2–5]. Although several risk factors have been identified (such as advanced age, comorbidity burden, and preexisting cognitive decline), the underlying pathophysiology of delirium remains poorly understood [1]. Therefore, it is necessary to explore and identify biomarkers associated with delirium, which may not only advance our understanding of delirium but also help clinicians evaluate delirium risk more accurately and design interventions accordingly to prevent the condition and improve the outcomes.

Stress hyperglycemia is prevalent among patients with acute illness and generally refers to the transient increase in blood glucose as a result of inflammation and neuroendocrine derangements during illness [6]. It has been reported that stress hyperglycemia is associated with poor prognosis in hospitalized patients [7–11]. However, few studies have previously examined the role of stress hyperglycemia in delirium. As elevated glucose levels can induce oxidative stress, compromise the blood-brain barrier, and cause subsequent neuronal damage that may lead to delirium [12, 13], we postulated that stress hyperglycemia may be a potential risk factor for delirium. Unfortunately, in clinical practice, there is currently no consensus on the definition of stress hyperglycemia. Most previous studies relied on admission glucose concentration to identify stress hyperglycemia, which cannot truly reflect the acute glycemic increase in patients with poor diabetes control.

Recently, stress hyperglycemia ratio (SHR), which combines admission glucose and the estimated average glucose derived from glycosylated hemoglobin (HbA1c), was proposed as a novel indicator of stress hyperglycemia [14]. As HbA1c reflects the mean glucose status over the preceding 3 months [15, 16] and is not easily affected by the onset of acute illness [17], it can be speculated that SHR could quantify stress hyperglycemia more accurately by controlling for background glycemia. Growing studies have suggested that the prognostic value of SHR is superior to admission glycemia [14, 18, 19]. However, to date, no evidence exists on the relationship between SHR and delirium.

In the present study, we therefore aimed to explore the association between SHR and delirium in older hospitalized patients. We hypothesized that patients with higher SHR levels would be more likely to experience delirium.

## Methods

### Study population

This study was conducted in the Department of Geriatrics (across four floors), West China Hospital of Sichuan University, between March 2016 and July 2017. Each floor is equipped with an average of 65 beds and receives internal medical patients aged 60 years or older. Eligible patients were aged ≥70 years and had an anticipated length of hospital stay of at least 3 days. Exclusion criteria included delirium on admission, inability to communicate due to severe deafness or severe dementia, a documented history of psychiatric illness, terminal conditions with a life expectancy of less than 6 months, and incomplete data. The study was approved by the Biomedical Research Ethics Committee of West China Hospital, Sichuan University, and conformed to the ethical guidelines of the Declaration of Helsinki. Written informed consent was obtained from all participants.

### Data collection

The following baseline demographic and clinical characteristics were collected by trained research nurses within 24 hours after admission: age, sex, body mass index, education levels, marriage status, smoking, alcohol consumption, type of admission, and history of diabetes mellitus. Baseline functional status was assessed by the Barthel Index for activities of daily living (ADL) [20]. Cognitive function was evaluated using the Short Portable Mental Status Questionnaire (SPMSQ), and errors ≥3 were defined as cognitive impairment [21]. The severity of comorbidities was measured with the Charlson Comorbidity Index (CCI) [22]. Visual acuity and hearing ability were evaluated using the Snellen eye chart and the whispered voice test, respectively. The severity of pain was measured by Wong-Baker faces pain rating scale, and a score ≥2 was defined as having pain [23]. Laboratory tests (routine blood tests, blood urea nitrogen, creatinine, and albumin) were conducted within 24 hours of admission.

### Assessment of stress hyperglycemia ratio

Venous blood samples were collected from all patients after an overnight fast within the first two days after admission. Fasting blood glucose (FBG) was measured using the hexokinase method (Cobas 8000, ROCHE, Germany), while HbA1c was measured by high-performance liquid chromatography (Tosoh G8). All measurements were performed by laboratory personnel blinded to patients’ clinical characteristics and outcomes. Hypoglycemia was defined as FBG <3.9 mmol/L [24, 25], and hyperglycemia as FBG >7.8 mmol/L [26]. And HbA1c ≥6.5% designated the presence of background hyperglycemia.

HbA1c was used to estimate the average blood glucose before admission using the following formula: estimated average glucose _(mmol/L)_ = (1.59 × HbA1c) - 2.59 [16]. Stress hyperglycemia ratio (SHR) was then calculated as the ratio of FBG to the estimated average glucose, which quantified the extent of acute elevation in blood glucose compared with the background glucose status.

### Outcome assessment

The main outcome measure was the development of delirium during hospitalization. Delirium was assessed using the Confusion Assessment Method (CAM), which is a widely used, standardized diagnostic tool for delirium with high sensitivity (94% to 100%), specificity (90% to 95%), and interrater reliability (0.70 to 1.00) [27]. The CAM diagnostic algorithm requires the presence of acute onset or fluctuating course, inattention, and either disorganized thinking or an altered level of consciousness to fulfill the criteria for delirium.

Research nurses were trained ahead in screening delirium to ensure high interrater and intrarater reliability (kappa ≥0.9). Then, all patients were screened for delirium by well-trained nurses within 24 h after admission and three times daily thereafter until discharge or for a maximum of 13 days. In addition, to minimize error and maximize reliability, experienced clinical researchers further independently assessed patients every 48 h. In case of any doubt, an expert panel was consulted to screen patients according to the Diagnostic and Statistical Manual of Mental Disorders, Fourth Edition criteria [28].

### Statistical analysis

Continuous data were reported as mean with standard deviation (SD) or median with interquartile range (IQR), while categorical data were expressed as numbers and percentages. Since there are no established thresholds for SHR and given the sample size included in this study, we divided participants into three groups according to SHR tertiles. Differences among tertiles were compared using one-way analysis of variance or Kruskal-Wallis test for continuous variables and χ^2^ test or Fisher’s exact test for categorical variables.

We calculated the incidence rate of delirium by SHR tertiles. Subsequently, multivariate Cox proportional hazards models were used to explore the association between SHR and delirium. SHR was first included in the Cox models as a continuous variable (per 0.1 increase) and then was entered as a categorical variable (tertile groups). The tertile with the lowest delirium incidence was defined as the reference group. We performed 3 models: model 1 adjusted for age and sex; model 2 included the same variables in model 1 as well as potential delirium risk factors with *P*<0.10 identified in the univariate analysis; and model 3 further adjusted for statin comedication after admission since previous studies have shown that statins may affect the development of delirium [29]. In addition, the restricted cubic spline was performed to evaluate the pattern and magnitude of the associations between SHR and delirium, with four knots (at the 5th, 35th, 65th, 95th percentiles) [30]. Furthermore, C statistics, net reclassification index (NRI), and integrated discrimination improvement (IDI) were calculated to evaluate the incremental predictive value of SHR beyond conventional risk factors.

As premorbid glycemia status might affect the relationship between blood glucose and outcomes [31, 32], subgroup analysis was conducted to test whether the association of SHR with delirium differs between patients with and without background hyperglycemia. In addition, some factors (i.e., age [33], infection [7], chronic kidney impairment [34], anemia [16], etc.) might influence the development of hyperglycemia or the values of HbA1c, which may potentially bias the link between SHR and delirium. Thus, additional stratified analyses were further used to assess the potential effect modification by these variables. Interactions between SHR levels and stratified factors were examined using the likelihood ratio test.

All statistical analyses were performed using Stata 15.0 (StataCorp, College Station, TX, USA) and R version 3.4.3 (R Foundation for Statistical Computing, Vienna, Austria). Two-sided values of *P*<0.05 were considered statistically significant.

## Results

### Baseline characteristics

**Fig. 1:**
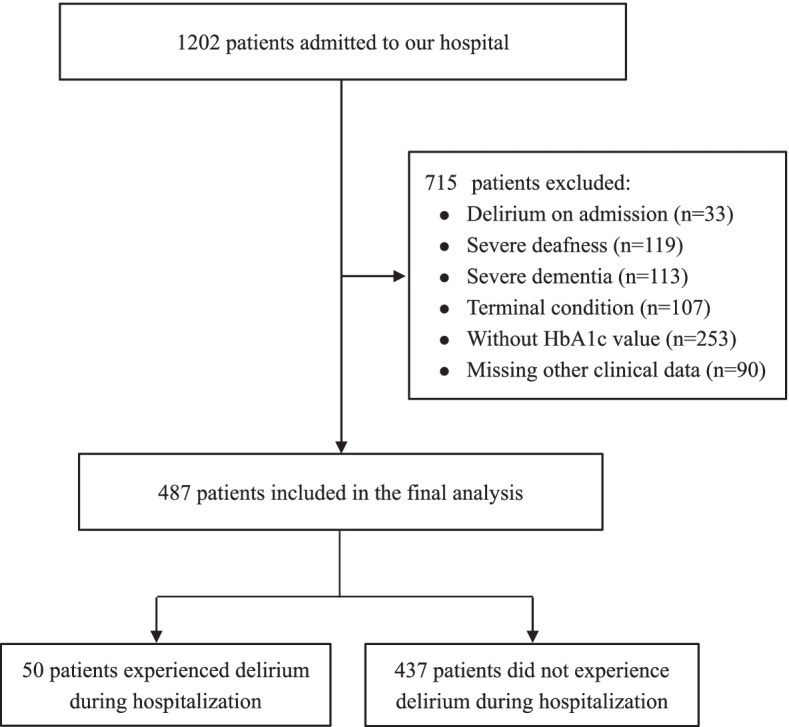
Flow chart of participants’ selection. HbA1c, glycosylated hemoglobin

During the study period, there were 1202 patients admitted to our hospital, and 715 patients were excluded (Fig. 1). Therefore, a total of 487 patients were included in the final analysis. The mean age was 83.0 ± 5.9 years, and 351 (72.0%) were male. The median value of SHR was 0.81 (IQR 0.72-0.96). All patients were divided into three groups based on SHR tertiles (tertile 1, <0.753; tertile 2, 0.753-0.895; and tertile 3 >0.895). Table 1 shows the baseline characteristics of patients by SHR tertiles. Patients in the highest tertile were more likely to have cognitive impairment and infection, had higher levels of blood glucose, white blood cells, blood urea nitrogen and creatinine, had lower estimated glomerular filtration rate, and lower ADL scores (all *P*<0.05, Table 1).

**Table 1:** Baseline characteristics of patients by tertiles of stress hyperglycemia ratio

**Characteristics**	**Total (*n*=487)**	**Tertile 1 (*n*=162)**	**Tertile 2 (*n*=162)**	**Tertile 3 (*n*=163)**	***P***
Age, mean (SD)	83.0 (5.9)	82.8 (5.6)	83.0 (5.9)	83.3 (6.2)	0.81
Male, n (%)	351 (72.0)	118 (72.8)	107 (66.0)	126 (77.3)	0.08
BMI, kg/m^2^, mean (SD)	22.8 (3.5)	22.5 (3.1)	23.2 (3.9)	22.9 (3.6)	0.17
Married, n (%)	405 (83.2)	142 (87.7)	133 (82.1)	130 (79.8)	0.15
High school and above, n (%)	337 (69.2)	112 (69.2)	110 (67.9)	115 (70.5)	0.92
Smoker, n (%)	180 (37.0)	59 (36.4)	50 (30.9)	71 (43.6)	0.06
Drinker, n (%)	99 (20.3)	27 (16.7)	34 (21.0)	38 (23.3)	0.32
Diabetes mellitus, n (%)	184 (37.8)	60 (37.0)	57 (35.2)	67 (41.1)	0.53
Emergency admission, n (%)	51 (10.5)	14 (8.6)	10 (6.2)	27 (16.6)	0.01
Blood glucose, mmol/L, mean (SD)	6.7 (3.0)	5.1 (1.2)	6.2 (1.6)	8.8 (3.9)	<0.001
Hypoglycemia, n (%)	8 (1.6)	8 (4.9)	0 (0.0)	0 (0.0)	<0.001
Hyperglycemia, n (%)	93(19.1)	5 (3.1)	20 (12.3)	68 (41.7)	<0.001
HbA1c, %, mean (SD)	6.4 (1.2)	6.5 (1.3)	6.4 (1.2)	6.4 (1.2)	0.51
SHR, median (IQR)	0.81 (0.72-0.96)	0.69 (0.64-0.72)	0.81 (0.78-0.85)	1.05 (0.96-1.22)	<0.001
WBC, ×10^9^/L, mean (SD)	6.7 (2.7)	6.3 (2.0)	6.5 (2.9)	7.3 (3.0)	0.004
BUN, mmol/l, mean (SD)	7.8 (5.7)	6.9 (3.4)	6.9 (4.2)	9.5 (8.1)	<0.001
Creatinine, umol/L, median (IQR)	82.0 (66.8-100.0)	78.5 (66.6-94.0)	78.0 (64.0-93.3)	89.0 (71.0-117.9)	0.001
Albumin, g/l, mean (SD)	39.0 (4.6)	39.0 (4.4)	39.5 (4.4)	38.5 (5.0)	0.14
eGFR, ml/min/1.73 m^2^, mean (SD)	73.9 (28.1)	77.1 (25.4)	77.0 (27.2)	67.6 (30.5)	0.003
Infection, n (%)	93 (19.1)	23 (14.2)	25 (15.4)	45 (27.6)	0.003
Pain, n (%)	257 (52.8)	78 (48.1)	93 (57.4)	86 (52.8)	0.25
Anemia, n (%)	133 (27.3)	37 (22.8)	40 (24.7)	56 (34.4)	0.04
Vision impairment, n (%)	168 (34.5)	48 (29.6)	59 (36.4)	61 (37.4)	0.28
Hearing impairment, n (%)	164 (33.7)	52 (32.1)	56 (34.6)	56 (34.4)	0.87
Cognitive impairment, n (%)	137 (28.1)	40 (24.7)	34 (21.0)	63 (38.7)	0.001
ADL, median (IQR)	85 (60-95)	90 (60-100)	90 (64-100)	75 (45-95)	<0.001
CCI, median (IQR)	1 (1-2)	1 (0-2)	1 (1-2)	1 (1-3)	0.10
Statin treatment after admission, n (%)	234 (48.0)	81 (50.0)	87 (53.7)	66 (40.5)	0.05
Hospitalization days, median (IQR)	18 (13-26)	17 (12-24)	18 (13-24)	18 (12-28)	0.38

*BMI* Body mass index, *HbA1c* Glycosylated hemoglobin, *SHR* Stress hyperglycemia ratio, *WBC* White blood cell, *BUN* Blood urea nitrogen, *eGFR* Estimated glomerular filtration rate, *ADL* Activities of daily living, *CCI* Charlson comorbidity index, *SD* Standard deviation, *IQR* Interquartile range, *eGFR* was calculated using the Modification of Diet in Renal Disease (MDRD) equation. Statin includes rosuvastatin, atorvastatin, and simvastatin

SHR tertiles: Tertile 1 <0.753, Tertile 2 0.753-0.895, Tertile 3 >0.895

### Association between SHR and delirium

Fifty (10.3%) patients experienced delirium during hospitalization, and the median time from admission to delirium occurrence was 3 days (IQR, 3-7 days). The second SHR tertile had the lowest delirium incidence (tertile 1 vs. tertile 2 vs. tertile 3, 8.6% vs. 4.9% vs. 17.2%, *P*=0.001, Table 2). The overall incidence rate of delirium was 9.1 per 1000 person-days. For SHR tertiles, the incidence rate of delirium was 7.7, 4.2, and 16.1 per 1000 person-days for patients in tertile 1, tertile 2, and tertile 3, respectively (Table 2).

**Table 2:** The overall incidence rate of delirium and incidence rate by SHR tertiles

	**Number of delirium (%)**	**Total person-days**	**Events per 1000 person-days (95% CI)**
Total (*n*=487)	50 (10.26)	5483	9.10 (6.90-12.00)
Tertile 1 (*n*=162)	14 (8.64)	1827	7.70 (4.60-12.90)
Tertile 2 (*n*=162)	8 (4.94)	1916	4.20 (2.10-8.30)
Tertile 3 (*n*=163)	28 (17.17)	1740	16.10 (11.20-23.20)

*SHR* Stress hyperglycemia ratio, *CI* Confidence interval

The results of the univariate analysis are presented in the Additional file 1 (Table A1). The SHR level was significantly higher in patients with delirium (median value, 0.91 vs. 0.80, *P*=0.03). Besides, patients with delirium were older, more likely to be smokers, had higher proportion of vision, hearing, and cognitive impairment, had higher white blood cell counts, lower albumin levels, higher comorbidity burden, lower ADL scores, and lower proportion of statin treatment than those without delirium (all *P*<0.05, Table A1).

**Fig. 2:**
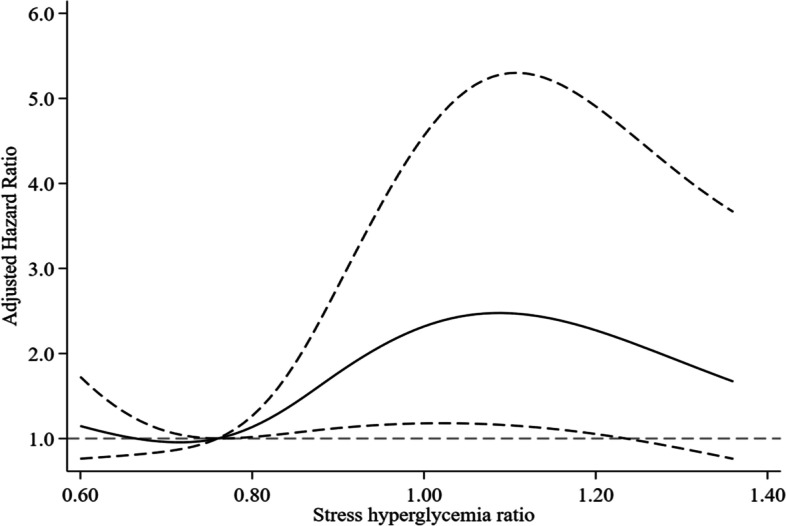
Multiple spline regression analyses to examine the relationship between SHR and delirium. The solid line represents hazard ratios and the dotted lines represent 95% confidence intervals. Reference is the 35th percentile of SHR (0.76). Hazard ratios for delirium were adjusted for the same variables as model 2 in Table 3. The lowest 5% and highest 5% of patients were not shown in the figure due to small sample sizes

In the multivariate analysis, after adjusting for potential confounders, we found that patients in the lowest and highest tertiles had a significantly higher risk of delirium than those in the second SHR tertile (model 2: HR 3.71, 95% CI 1.45-9.51; and HR 2.97, 95% CI 1.29-6.81, respectively). In the sensitivity analysis further adjusted for statin treatment, similar results were observed (model 3, Table 3). The multiple-adjusted spline regression model revealed a nonlinear association between SHR levels and delirium (*P* for nonlinearity=0.04, Fig. 2).

**Table 3:** Multivariate analyses between SHR and delirium

**Groups**	**Model 1**		**Model 2**		**Model 3**	
	**HR (95%CI)**	***P***	**HR (95%CI)**	***P***	**HR (95%CI)**	***P***
**All patients**						
SHR, per 0.1 increase	1.06 (0.97-1.16)	0.19	0.96 (0.87-1.05)	0.38	0.96 (0.87-1.06)	0.43
SHR tertiles						
T1 (<0.753)	1.89 (0.79-4.50)	0.15	3.71 (1.45-9.51)	0.01	3.33 (1.28-8.73)	0.01
T2 (0.753-0.895)	Ref		Ref		Ref	
T3 (>0.895)	3.66 (1.67-8.05)	0.001	2.97 (1.29-6.81)	0.01	2.72 (1.17-6.36)	0.02
**HbA1c <6.5%**						
SHR, per 0.1 increase	1.12 (0.98-1.28)	0.11	1.00 (0.86-1.18)	0.96	1.01 (0.87-1.18)	0.88
SHR tertiles						
T1 (<0.754)	1.39 (0.48-4.00)	0.55	3.65 (1.11-11.97)	0.03	3.34 (0.97-11.49)	0.05
T2 (0.754-0.893)	Ref		Ref		Ref	
T3 (>0.893)	2.94 (1.16-7.45)	0.02	3.13 (1.13-8.72)	0.03	2.96 (1.04-8.41)	0.04

*SHR* Stress hyperglycemia ratio, *HbA1c* Glycosylated hemoglobin, *HR* Hazard ratio, *CI* Confidence interval

Model 1: adjusted for age and sex; Model 2: adjusted for model 1 + white blood cell, albumin, smoking, vision impairment, hearing impairment, cognitive impairment, activities of daily living, and Charlson comorbidity index; Model 3: adjusted for model 2 + statin treatment after admission

### Incremental predictive value of SHR

The performance of models with SHR to predict delirium is shown in Table 4. Adding SHR to the conventional model did not significantly improve discriminatory power but did significantly improve risk reclassification, as observed by the continuous NRI of 0.39 (95% CI 0.09-0.56) and IDI of 0.07 (95% CI 0.01-0.14).

**Table 4:** Reclassification and discrimination statistics for risk of delirium by SHR

	**C statistic**		**NRI (continuous)**		**IDI**	
	**Estimate (95% CI)**	***P***	**Estimate (95% CI)**	***P***	**Estimate (95% CI)**	***P***
Conventional model	0.93 (0.90-0.95)		Ref		Ref	
Conventional model + SHR (tertiles)	0.93 (0.91-0.96)	0.19	0.39 (0.09-0.56)	0.01	0.07 (0.01-0.14)	0.03

*SHR* Stress hyperglycemia ratio, *NRI* Net reclassification improvement, *IDI* Integrated discrimination index, *CI* Confidence interval

Conventional model: age, sex, white blood cell, albumin, smoking, vision impairment, hearing impairment, cognitive impairment, activities of daily living, and Charlson comorbidity index

### Subgroup analyses

The characteristics of patients with HbA1c ≥6.5% vs. HbA1c <6.5% are detailed in the Additional file 1 (Table A2). In patients with HbA1c ≥6.5%, there was no significant association between SHR and delirium, regardless of whether SHR was a continuous or categorical variable (all *P*>0.05). In contrast, in the subgroup of HbA1c <6.5%, patients with delirium had significantly higher SHR levels than those without delirium (median value, 0.92 vs. 0.80, *P*=0.04, Table A2). After adjustment for covariates in model 2, we found that the first and third SHR tertiles in patients with HbA1c <6.5% were independently related to delirium, and the adjusted HR was 3.65 for the first vs. the second tertile (95% CI 1.11-11.97) and 3.13 for the third vs. the second tertile (95% CI 1.13-8.72, Table 3). A similar tendency of association between SHR and delirium was observed after further adjusting for statin in model 3 (Table 3).

**Fig. 3:**
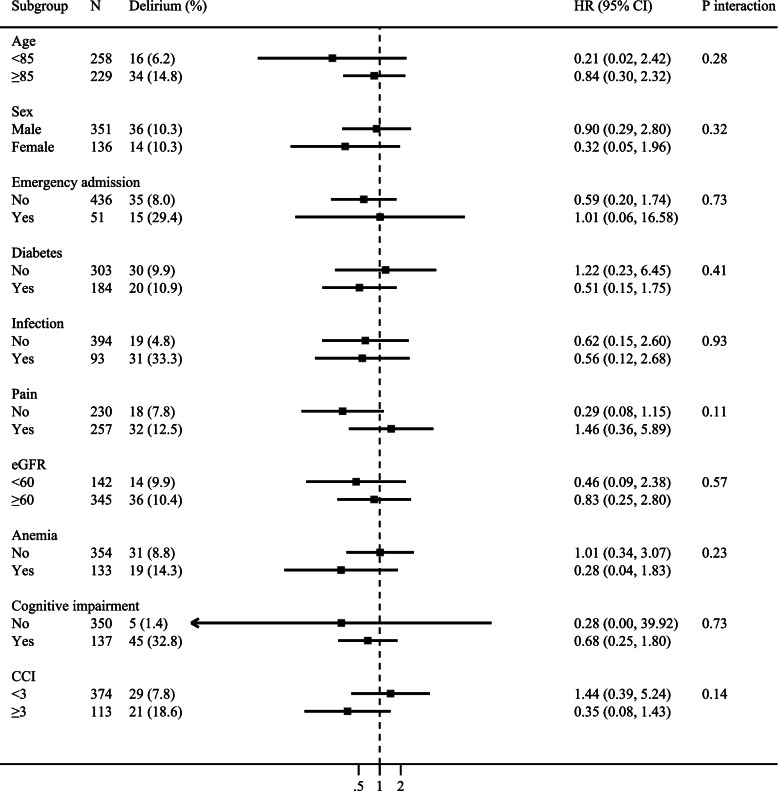
Stratified analyses to identify variables that may modify the association between SHR and delirium. Hazard ratios were adjusted for the same variables as model 2 in Table 3, except for the stratified variable. eGFR, estimated glomerular filtration rate; CCI, Charlson comorbidity index; HR, hazard ratio; CI, confidence interval

Additional stratified analyses showed that the relationship between SHR and delirium was not changed by age, sex, emergency admission, previous diabetes, infection, pain, renal dysfunction, anemia, cognitive impairment, or comorbidity burden (all *P*-interaction >0.05, Fig. 3).

## Discussion

In this study, we found that both lower and higher SHRs were significantly associated with an increased risk of delirium in older hospitalized patients. Subgroup analyses indicated that the associations of SHR with delirium were more apparent in patients with HbA1c <6.5%. The inclusion of SHR to conventional risk factors improved risk prediction for delirium. These results highlight the need to consider admission SHR when evaluating delirium risk, especially in those with HbA1c <6.5%.

Although we used the well-validated assessment tool to diagnose delirium and the assessors were heavily trained to accurately apply the assessment tool before the study, the incidence of delirium in our study was lower (10.3%). And a recent meta-analysis of older medical inpatients reported that delirium was present in up to 23% [35]. Compared to those previous studies mostly conducted in Western European medical populations [35], our population might be healthier and less impaired than similar internal medicine populations in Western European countries, as indicated by lower comorbidities burden, better functional status and cognitive function. Also of note, the culture difference might play a role as older adults in China have close relationships with their family members, who are often involved in their care and may exert positive effects in reducing delirium risk [36]. These might help explain the relatively lower incidence of delirium in this study.

Glucose dysregulation has been identified as a risk factor for delirium [2, 37], but few studies have investigated the link between stress hyperglycemia and delirium. Recent studies on surgical patients reported that perioperative stress-induced hyperglycemia was positively related to postoperative delirium [38–40], which was partly in line with our results. However, most of the aforementioned studies utilized absolute glycemia (i.e., random or fasting glucose) to diagnose stress hyperglycemia without considering the background glucose. In clinical practice, hyperglycemia in a hospitalized patient may result not only from acute stress but also from chronic poor control of diabetes [14]. Unlike previous studies that relied mainly on absolute hyperglycemia, we chose an easily available indicator of relative hyperglycemia, measured as SHR, to assess the glycemic fluctuations during acute stress. By combining the acute and premorbid glycemia levels, SHR could identify and quantify stress hyperglycemia more accurately. Increasing evidence has shown that SHR is associated with unfavorable outcomes in various diseases [14, 18, 19, 41]. However, whether SHR is related to delirium remains unclear. Our study added evidence on the association between SHR and delirium, suggesting that SHR may provide important predictive information for delirium.

Contrary to our original hypothesis, we found that the relationship between SHR and delirium was nonlinear, and the second SHR tertile had the lowest delirium incidence, which might be attributed to the potential protective effect of stress hyperglycemia. Stress hyperglycemia has been suggested as a survival response that provides the brain with essential fuel at the time of stress [7]. Mild-to-moderate stress hyperglycemia may protect against pathological insults by reducing apoptosis, favoring angiogenesis, and increasing plasticity [7]. In contrast, attempts to interfere with this response may contribute to brain energy crisis [42]. Multiple studies have indicated that even mild hypoglycemia can cause alterations in brain function and neurocognitive performance [43, 44]. In our study, we noted that the first SHR tertile had a significantly lower glycemic level than the other two tertiles (Table 1). Further analysis found that the prevalence of hypoglycemia (<3.9 mmol/L) was highest in the first tertile (tertile 1 vs. tertile 2 vs. tertile 3, 4.9% vs. 0.0% vs. 0.0%, *P*<0.001), in which delirium incidence was higher. However, hypoglycemia may account only partially for the high delirium rate in the first SHR tertile, since 95% of delirium patients did not have hypoglycemia. It might be possible that some hypoglycemic episodes were not recorded, as glucose was only measured once. Therefore, the contribution of hypoglycemia to delirium may be underestimated and needs further research using continuous glucose monitoring.

Stress hyperglycemia is caused by inflammatory and neurohormonal derangements that occur during acute illness [6, 7]. Thus, a higher SHR may reflect more severe baseline inflammation and neuroendocrine response, both of which are reported to be involved in the development of delirium [2, 45]. Besides, stress hyperglycemia may directly contribute to negative outcomes by inducing oxidative stress and endothelial dysfunction [6]. Several studies have found that increased glucose concentrations can promote the release of proinflammatory cytokines, damage the blood-brain barrier, and induce neuroinflammation [12, 13], which may ultimately cause disturbances in the neuronal network and resultant delirium [46]. Our results concerning the relationship between higher SHR and delirium might be supported by these findings.

It is worth noting that stress hyperglycemia usually occurs in the first few days after insult and may resolve spontaneously as acute illness or stress dissipates [6], which may potentially affect the association between SHR and delirium. The timing, severity, and duration of stress hyperglycemia may vary with patients’ glucose tolerance, type and severity of disease, and stage of illness [6]. Although delirium in this study occurred relatively early (median time 3 days, IQR 3-7 days), neither the severity and stage of patients’ acute disease nor the inflammatory cytokines and neurohormones (such as catecholamines and cortisol) that can reflect the degree of stress was dynamically assessed. At the same time, blood glucose was measured only on admission without continuous monitoring, and the temporal profile of stress hyperglycemia (or SHR variability) was unclear. Whether SHR variability affects the development of delirium remains unknown and requires further investigation.

Premorbid glycemia status can modify the correlation between admission glucose and patient prognosis [31, 32], so it seems necessary to conduct a subgroup analysis based on background hyperglycemia. In our study, although no significant interaction was observed between SHR and background hyperglycemia (*P*=0.28), we found that the first and third SHR tertiles were associated with delirium, yet only in patients with HbA1c <6.5% (Table 3). This may be explained by the findings that patients with premorbid hyperglycemia tolerated high-range fluctuations in glucose levels better than those without premorbid hyperglycemia [47–49]. In addition, chronic premorbid hyperglycemia can result in cellular conditioning that might exert a protective effect against the adverse consequences of stress hyperglycemia [6, 50]. For example, acute stress may induce the expression of glucose transporters (GLUTs) in cell membranes, causing uncontrolled entry of glucose into cells and resulting in glucose toxicity [6]. However, chronic exposure to hyperglycemia preferentially downregulates GLUT-1 and GLUT-3, which may protect against glucose influx during acute stress [6, 50]. Moreover, cellular adaptation to chronic hyperglycemia might be protective by reducing the production of superoxide [51]. Nevertheless, we found that the median SHR level in patients with HbA1c ≥6.5% and < 6.5% were almost the same and also the incidence rates of delirium in the tertiles (Table A2). The lack of relationship between SHR and delirium in those with HbA1c ≥6.5% may be an artifact of the small sample size since only 19 patients experienced delirium in this subgroup, which may limit the statistical power and raise the chance of false negatives [52]. Future studies with a larger sample size are required to validate our results.

The strengths of this study include that we used a simple, easily accessible indicator and identified its potential clinical usefulness in predicting delirium, especially in those with HbA1c <6.5%. Since glucose and HbA1c tests are widely available, SHR can easily be calculated in a busy clinical setting without needing complicated formulas and additional costs. However, this study has some limitations. First, it was a single-center observational study, and therefore, conclusions about causality cannot be drawn. Second, potential selection bias may be introduced, as patients without HbA1c values were excluded. Third, the severity or stage of patients’ acute illness and some therapeutic interventions (such as corticosteroids, parental and enteral nutrition) that may influence the development of hyperglycemia [6] were not collected, which may potentially bias the relationship between SHR and delirium. Fourth, SHR was not dynamically measured, and the impact of SHR variability on delirium remains undefined. Finally, we did not collect sufficient data on treatment options because treatment varied widely from patient to patient due to different comorbidities. As many variables associated with delirium were not available in this study, residual confounding cannot be excluded. Hence, SHR may be a surrogate for other factors not studied. Future larger prospective multicenter studies are warranted to confirm our findings.

## Conclusions

Both lower and higher SHR were associated with an increased risk of delirium in older hospitalized patients. SHR may serve as a promising marker to identify patients with a higher risk of delirium. Incorporating measures of SHR might have the potential clinical utility to aid delirium risk stratification. Further preclinical and clinical studies are needed to verify our results and to elucidate the mechanisms underlying SHR and delirium.

## Supplementary Information

**Additional file 1:** **Table A1.** Univariate analysis to identify potential risk factors associated with delirium. **Table A2.** Comparison of clinical characteristics between delirium and non-delirium patients, stratified by the presence/absence of background hyperglycemia.

## Data Availability

The datasets generated and/or analyzed during the current study are not publicly available as individual privacy could be compromised but are available from the corresponding author on reasonable request.

## Abbreviations

SHR: Stress hyperglycemia ratio
FBG: Fasting blood glucose
HbA1c: Glycosylated hemoglobin
HR: Hazard ratio
CI: Confidence interval
ADL: Activities of daily living
SPMSQ: The Short Portable Mental Status Questionnaire
CCI: The Charlson Comorbidity Index
CAM: Confusion assessment method
SD: Standard deviation
IQR: Interquartile range
NRI: Net reclassification index
IDI: Integrated discrimination improvement
GLUT: Glucose transporter
