# Supplementary material for: Association between stress hyperglycemia ratio and delirium in older hospitalized patients: a cohort study
Source: BMC Geriatr. 2022 Apr 4;22:277. doi: 10.1186/s12877-022-02935-6 (PMC8978391; doi:10.1186/s12877-022-02935-6)
Supplement: Supplementary file 1 — Additional file 1: Table A1. Univariate analysis to identify potential risk factors associated with delirium. Table A2. Comparison of clinical characteristics between delirium and non-delirium patients, stratified by the presence/absence of background hyperglycemia. [file 12877_2022_2935_MOESM1_ESM.docx]

**Table A1.** Univariate analysis to identify potential risk factors associated with delirium.

| **Characteristics** | **Delirium (n=50)** | **Non-delirium (n=437)** | ***P*** |
| --- | --- | --- | --- |
| Age, mean (SD) | 85.9 (4.9) | 82.7 (5.9) | <0.001 |
| Male, n (%) | 36 (72.0) | 315 (72.1) | 0.99 |
| BMI, kg/m^2^, mean (SD) | 22.2 (3.9) | 22.9 (3.5) | 0.17 |
| Married, n (%) | 35 (70.0) | 370 (84.7) | 0.01 |
| High school and above, n (%) | 31 (62.0) | 306 (70.0) | 0.16 |
| Smoker, n (%) | 28 (56.0) | 152 (34.8) | 0.003 |
| Drinker, n (%) | 10 (20.0) | 89 (20.4) | 0.95 |
| Diabetes mellitus, n (%) | 20 (42.0) | 164 (37.5) | 0.73 |
| Blood glucose, mmol/L, mean (SD) | 7.3 (2.8) | 6.7 (3.0) | 0.17 |
| HbA1c, %, mean (SD) | 6.6 (1.3) | 6.4 (1.2) | 0.41 |
| SHR, median (IQR) | 0.91 (0.74-1.05) | 0.80 (0.72-0.95) | 0.03 |
| Tertile 1 (<0.753), n (%) | 14 (28.0) | 148 (33.9) | 0.001 |
| Tertile 2 (0.753-0.895), n (%) | 8 (16.0) | 154 (35.2) |  |
| Tertile 3 (>0.895), n (%) | 28 (56.0) | 135 (30.9) |  |
| WBC, ×10^9^/L, mean (SD) | 7.4 (2.7) | 6.6 (2.7) | 0.05 |
| BUN, mmol/l, mean (SD) | 9.0 (8.0) | 7.6 (5.4) | 0.26 |
| Creatinine, umol/L, median (IQR) | 78.0 (58.5-93.3) | 82.0 (67.0-102.0) | 0.11 |
| Albumin, g/l, mean (SD) | 35.5 (5.4) | 39.4 (4.4) | <0.001 |
| Vision impairment, n (%) | 28 (56.0) | 140 (32.0) | 0.001 |
| Hearing impairment, n (%) | 26 (52.0) | 138 (31.6) | 0.004 |
| Cognitive impairment, n (%) | 45 (90.0) | 92 (21.1) | <0.001 |
| ADL, median (IQR) | 40 (25-55) | 90 (65-100) | <0.001 |
| CCI, median (IQR) | 2 (1-4) | 1 (1-2) | <0.001 |
| Statin treatment after admission, n (%) | 17 (34.0) | 217 (49.7) | 0.04 |
| Hospitalization days, median (IQR) | 24.5 (15.8-31) | 17 (12-25) | <0.001 |

BMI, body mass index; HbA1c, glycosylated hemoglobin; SHR, stress hyperglycemia ratio; WBC, white blood cell; BUN, blood urea nitrogen; ADL, activities of daily living; CCI, Charlson comorbidity index; SD, standard deviation; IQR, interquartile range. Statin includes rosuvastatin, atorvastatin, and simvastatin.

**Table A2.** Comparison of clinical characteristics between delirium and non-delirium patients, stratified by the presence/absence of background hyperglycemia.

| **Characteristics** | **HbA1c ≥6.5% (n=164)** | | ***P*** | **HbA1c <6.5% (n=323)** | | ***P*** |
| --- | --- | --- | --- | --- | --- | --- |
|  | **Delirium** | **Non-delirium** |  | **Delirium** | **Non-delirium** |  |
| No. (%) | 19 (11.6) | 145 (88.4) |  | 31 (9.6) | 292 (90.4) |  |
| Age, mean (SD) | 86.2 (4.3) | 82.1 (6.4) | 0.001 | 85.9 (5.4) | 83.0 (5.7) | 0.01 |
| Male, n (%) | 12 (63.2) | 107 (73.8) | 0.33 | 24 (77.4) | 208 (71.2) | 0.47 |
| BMI, kg/m^2^, mean (SD) | 23.5 (4.0) | 23.0 (3.5) | 0.58 | 21.4 (3.7) | 22.9 (3.5) | 0.03 |
| Married, n (%) | 13 (68.4) | 126 (86.9) | 0.05 | 22 (71.0) | 244 (83.6) | 0.08 |
| High school and above, n (%) | 12 (63.2) | 103 (71.0) | 0.48 | 19 (8.6) | 203 (69.5) | 0.35 |
| Smoker, n (%) | 11 (57.9) | 54 (37.2) | 0.08 | 17 (54.8) | 98 (33.6) | 0.02 |
| Drinker, n (%) | 3 (15.8) | 33 (22.8) | 0.77 | 7 (22.6) | 56 (19.2) | 0.65 |
| Diabetes mellitus, n (%) | 18 (94.7) | 110 (75.9) | 0.08 | 2 (6.5) | 54 (18.5) | 0.09 |
| Blood glucose, mmol/L, mean (SD) | 9.0 (3.2) | 8.7 (4.2) | 0.73 | 6.2 (1.9) | 5.6 (1.2) | 0.14 |
| HbA1c, %, mean (SD) | 7.9 (1.2) | 7.7 (1.3) | 0.67 | 5.8 (0.4) | 5.8 (0.4) | 0.88 |
| SHR, median (IQR) | 0.91 (0.70-1.09) | 0.81 (0.70-0.96) | 0.37 | 0.92 (0.74-1.04) | 0.80 (0.73-0.94) | 0.04 |
| Tertile 1, n (%) | 6 (31.6) | 49 (33.8) | 0.30 | 8 (25.8) | 99 (33.9) | 0.02 |
| Tertile 2, n (%) | 4 (21.1) | 51 (35.2) |  | 6 (19.4) | 103 (35.3) |  |
| Tertile 3, n (%) | 9 (47.4) | 45 (31.0) |  | 17 (54.8) | 90 (30.8) |  |
| WBC, ×10^9^/L, mean (SD) | 8.4 (3.1) | 7.1 (2.9) | 0.07 | 6.8 (2.4) | 6.4 (2.6) | 0.37 |
| BUN, mmol/l, mean (SD) | 10.4 (11.6) | 7.9 (4.3) | 0.36 | 8.0 (4.6) | 6.8 (2.4) | 0.63 |
| Creatinine, umol/L, median (IQR) | 79.0 (65.0-99.0) | 87.0 (69.0-110.0) | 0.50 | 74.0 (53.0-90.0) | 80.0 (66.0-98.0) | 0.12 |
| Albumin, g/l, mean (SD) | 36.9 (5.2) | 39.4 (4.2) | 0.02 | 34.7 (5.4) | 39.4 (4.4) | <0.001 |
| Vision impairment, n (%) | 13 (68.4) | 42 (29.0) | 0.001 | 15 (48.4) | 98 (33.6) | 0.10 |
| Hearing impairment, n (%) | 10 (52.6) | 46 (31.7) | 0.07 | 16 (51.6) | 92 (31.5) | 0.02 |
| Cognitive impairment, n (%) | 17 (89.5) | 28 (19.3) | <0.001 | 28 (90.3) | 64 (21.9) | <0.001 |
| ADL, median (IQR) | 40 (25-55) | 90 (63-100) | <0.001 | 40 (25-55) | 90 (65-100) | <0.001 |
| CCI, median (IQR) | 3 (2-4) | 2 (1-3) | 0.003 | 2 (1-3) | 1 (0-2) | 0.003 |
| Statin treatment after admission, n (%) | 7 (36.8) | 81 (55.9) | 0.12 | 10 (32.3) | 136 (46.6) | 0.14 |
| Hospitalization days, median (IQR) | 28 (20-37) | 19 (14-26) | <0.001 | 22 (14-28) | 16 (12-24) | 0.05 |

HbA1c, glycosylated hemoglobin; BMI, body mass index; SHR, stress hyperglycemia ratio; WBC, white blood cell; BUN, blood urea nitrogen; ADL, activities of living; CCI, Charlson comorbidity index; SD, standard deviation; IQR, interquartile range. Statin includes rosuvastatin, atorvastatin, and simvastatin.
